# Expression of inflammation-related genes is associated with adipose tissue location in horses

**DOI:** 10.1186/1746-6148-9-240

**Published:** 2013-12-02

**Authors:** Lien Bruynsteen, Tim Erkens, Luc J Peelman, Richard Ducatelle, Geert PJ Janssens, Patricia A Harris, Myriam Hesta

**Affiliations:** 1 Department of Nutrition, Genetics and Ethology, Faculty of Veterinary Medicine, Ghent University, Heidestraat 19, Merelbeke 9820, Belgium; 2 Department of Pathology, Bacteriology and Avian Diseases, Faculty of Veterinary Medicine, Ghent University, Salisburylaan 133, Merelbeke B-9820, Belgium; 3 WALTHAM Centre for Pet Nutrition, Freeby Lane, Waltham-On-The-Worlds, Melton Mowbray, Leicestershire LE14 4RT, UK

**Keywords:** RT-qPCR, Equine adipose tissue, mRNA expression, Inflammation

## Abstract

**Background:**

In humans, adipose tissue (AT) originating from different depots shows varying gene expression profiles. In horses, the risk of certain metabolic disorders may also be influenced by the impact of specific AT depots. Macrophage infiltration in human and rat AT is considered to be a source of inflammatory changes. In horses, this relationship has not been extensively studied yet. Reverse transcription quantitative real-time polymerase chain reaction (RT-qPCR), a useful method to evaluate differences in mRNA expression across different tissues, can be used to evaluate differences between equine AT depots. For a correct interpretation of the RT-qPCR results, expression data have to be normalized by the use of validated reference genes. The main objectives of this study were to compare mRNA expression of inflammation-related genes, as well as adipocyte morphology and number between different equine AT depots; and in addition, to investigate the presence of antigen presenting cells in equine AT and any potential relationship with adipokine mRNA expression.

**Results:**

In this study, the mRNA expression of inflammation-related genes (leptin, chemokine ligand 5, interleukin 1β, interleukin 6, interleukin 10, adiponectin, matrix metalloproteinase 2, and superoxide dismutase 2) and candidate reference gene stability was investigated in 8 different AT depots collected from the nuchal, abdominal (mesenteric, retroperitoneal, and peri-renal) and subcutaneous (tail head and loin) AT region. By using GeNorm analysis, *HPRT1, RPL32,* and *GAPDH* were found to be the most stable genes in equine AT. The mRNA expression of leptin, chemokine ligand 5, interleukin 10, interleukin 1β, adiponectin, and matrix metalloproteinase 2 significantly differed across AT depots (P < 0.05). No significant AT depot effect was found for interleukin 6 and superoxide dismutase 2 (P > 0.05). Adipocyte area and number of antigen presenting cells per adipocyte significantly differed between AT depots (P < 0.05).

**Conclusions:**

Adipose tissue location was associated with differences in mRNA expression of inflammation-related genes. This depot-specific difference in mRNA expression suggests that the overall inflammatory status of horses could be partially determined by the relative proportion of the different AT depots.

## Background

Adipose tissue (AT) can be divided into brown and white AT [1]. The latter is now recognized as being more than an energy storage site. It is accepted as a highly active metabolic and endocrine organ [2,3] comprising different cell types (adipocytes, pre-adipocytes, endothelial cells, fibroblasts and macrophages) [4] that actively secrete proteins involved in the regulation of energy, as well as neuroendocrine, autonomic, and immune functions [5]. These different cell types may contribute to the secretion of the pro-inflammatory cytokines tumor necrosis factor alpha (TNF-α), interleukin 1 (IL-1), interleukin 6 (IL-6), chemokine ligand 5 (CCL5), and anti-inflammatory cytokine interleukin 10 (IL-10), as well as hormones such as resistin, leptin, and adiponectin that are involved in the inflammatory response and insulin sensitivity [5-7]*.*

The AT also secretes matrix metalloproteinases (MMPs, e.g. MMP-2 or gelatinase A, MMP-9 or gelatinase B [8], and MMP-1,3,7, [9]) which have a functional role in the development of the AT [10] and are important for the extracellular matrix remodelling, which occurs during obesity-mediated AT formation, at least in mice [11].

A study by Fain and colleagues [12] in obese women revealed that over 90% of the adipokine release by AT, except for adiponectin and leptin, could be attributed to non-fat cells. When excessive amounts of AT are deposited, inflammatory markers in the circulation can rise as result of the adipokine secreting ability of AT. Cinti and colleagues [13] demonstrated that > 90% of all macrophages in white AT of obese mice and humans were localized around dead adipocytes, forming crown-like structures (CLS). Vick and colleagues [14] first demonstrated an association between obesity and increased inflammatory markers (TNF-α and IL-1) in horses, although age was also an important and possible confounding factor. Currently, there is some controversy whether obesity in horses is or is not associated with low grade inflammation [14-16] and there is no evidence whether CLS do or do not form in the obese horse.

Adipocyte size is positively correlated with frequency of adipocyte death, macrophage numbers, as well as CLS in visceral and subcutaneous (SC) depots in mice [13,17], and leptin mRNA expression in humans and cattle [18,19]. To the authors’ knowledge, size of adipocytes originating from different horse AT regions has not been previously reported.

The specific site of AT deposition is clinically very important. Humans with a higher accumulation of visceral fat are at a higher risk for the development of obesity-related metabolic disorders [20]. Similarly in horses, it has recently been demonstrated that expression of glucose transporters was influenced by AT location in insulin sensitive and insulin resistant individuals [21]. It has also been suggested in equidae that AT distributed specifically on the crest of the neck could indicate or contribute to hyperinsulinemia, insulin resistance (IR), and/or an increased risk for laminitis [22,23]. Therefore, clinical interest on AT in this region is increased in horses [22,24]. Burns and coworkers [24] found no differences in pro-inflammatory cytokine IL-1β and IL-6 mRNA expression between insulin resistant and insulin sensitive horses. Higher mRNA concentrations of these two cytokines, however, were found in the nuchal ligament AT compared to the other AT depots sampled in that study.

Our hypothesis was that mRNA expression of inflammation-related genes varied across AT depots. Therefore, the first aim of this study was to compare adipocyte size and mRNA expression between different equine AT depots with special interest in the nuchal AT region. The second aim was to investigate the presence of antigen presenting cells in equine AT and any potential relationship with adipokine mRNA expression.

## Results and discussion

### Animals

A variety of different breeds was chosen for this study (Table 1). Average age was 14 ± 7 years.

**Table 1:** Information on the horses involved in this study

**Horse**	**Breed**	**Age (years)**	**Nutritional status**
1	German riding horse	16	overweight to obese
2	Dutch riding horse	20	normal
3	Belgian riding horse	1	normal
4	French riding pony, breed Haflinger	17	obese
5	Selle Français	10	normal
6	French Thouroughbred	12	normal
7	Royal Dutch Sport Horse (KWPN)	11	normal
8	Belgian trotter	3	normal
9	Belgian Warmblood (BWP)	21	overweight to obese
10	Dutch riding horse	25	overweight to obese
11	Dutch riding horse	11	overweight to obese
12	French trotter	15	overweight to obese

### Blood analysis

Average glucose, insulin, and leptin levels were 106 ± 16 mg/dl, 5.9 ± 0.9 mU/l, and 3.3 ± 1.4 ng/ml respectively.

### Candidate reference gene selection and GeNorm analysis

To study depot-related variation in mRNA expression in AT, a very sensitive and specific technique is required, such as RT-qPCR [25], because of the low yield of mRNA isolated from AT [26]. However, before comparing mRNA expression profiles across samples, correction for variables such as quality and quantification of the starting material and enzymatic efficiencies must be carried out [26-28]. Consequently, the need for accurate data normalization is crucial [29]. In human AT, obesity and type 2 diabetes can exert a detectable influence on reference gene expression in SC and visceral fat depots [30]. This demonstrates that the expression level of reference genes is influenced by body region and health status of the test subject.

The efficiency of each RT-qPCR run was calculated from a relative standard curve based on a 5-point 5-fold cDNA dilution series, and ranged between 93 and 102.5%. Linear correlation coefficients varied between 0.996 and 0.999.

One sample from the neck region, two from the SC region, and four from the abdominal region showed consistently higher transcription levels (Cq value: the fractional PCR cycle at which the fluorescent signal significantly rises above the background signal [31]) compared to the other samples. Amplification problems were considered to be the cause as the RNA quantity and quality was comparable to the other samples of the same region (Experion analysis). These 7 AT samples were therefore excluded from further analysis. Transcription levels across all AT studied were almost similar for *ACTB, GAPDH,* and *RPL32*, which had higher Cq values than *HPRT1, SDHA,* and *TUBA4A*. The raw gene expression data from the genes of interest were normalised using the geometric mean of the most stable candidate reference genes *GAPDH, HPRT1,* and *RPL32*.

### Depot-specific mRNA expression

The present study investigated the AT depot related mRNA expression of inflammation-related genes in horses of different breeds, different ages, and with varying body condition or nutritional status. Adipokine expression was primarily studied at the transcription level, which does not necessarily reflect the protein level and/or its activity. It should be mentioned that AT is made up of multiple cell types [4]. The aim of mRNA expression was not to examine the individual cell populations, but to consider AT as a whole [12].

Leptin mRNA expression was significantly higher in the three neck samples compared to the mesenteric AT samples (Neck (N) ¼, N ½, N ¾: P = 0.034, 0.008, and 0.015 respectively). In contrast, CCL5 and IL-10 showed significantly lower mRNA expression in the nuchal AT compared to the mesenteric AT (N ¼, N ½, N ¾; P = 0.009, 0.009, 0.019 for CCL5, and 0.032, 0.008, 0.009 for IL-10 respectively). A significant lower expression of adiponectin mRNA was found in the tail head AT region compared to the nuchal AT (N ¼, N ½, N ¾: P = 0.010, 0.014, and 0.004 respectively), retroperitoneal (P = 0.003), and peri-renal AT region (P = 0.009). Pro-inflammatory cytokine IL-1β mRNA expression was significantly lower in the loin AT compared to the mesenteric and peri-renal AT (P = 0.004). A trend for lower mRNA expression was found in the retroperitoneal AT (P = 0.074). The MMP2 mRNA expression was significantly lower in the peri-renal region compared to AT originating from N ¼ (P = 0.019), N ½ (P = 0.006), tail head (P = 0.004) and loin region (P = 0.008). Mesenteric AT had a significantly lower MMP2 mRNA expression compared to N ½ (P < 0.001), tail head (P = 0.004) and loin AT (P = 0.001). Retroperitoneal AT had a significantly lower MMP2 mRNA expression compared to the loin AT (P = 0.014). Interleukin 6 tended to have a higher mRNA expression in the N ½ AT compared to mesenteric AT (P = 0.073). No significant AT depot effect was found for superoxide dismutase (SOD) 2.

A correlation was found between plasma leptin and insulin concentrations (P = 0.035; r = 0.610). There was also a correlation between IL-6 and IL-1β in the nuchal AT region (N ¼, N ½, and N ¾: P = 0.004, 0.001, 0.003; r = 0.756, 0.827, 0.782 respectively) and the tail head region (P = 0.007; r = 0.734). Higher leptin mRNA expression in the nuchal AT region compared to the mesenteric AT suggests that nuchal AT may contribute proportionally more to the overall leptin concentration in the horse. The strong correlation between leptin concentration and degree of IR [32] supports the hypothesis that enlarged nuchal AT indeed is an important risk factor for IR [22,23]. In a study of Liburt and coworkers, decreased IL-6 mRNA in nuchal AT was associated with increased insulin sensitivity [33]. Higher nuchal AT IL-6 mRNA expression together with a significant correlation between the expression of IL-6 and IL-1β in the nuchal AT depot, could indicate that in horses, the nuchal AT depot is an important contributor for gene expression of these pro-inflammatory markers whereas in humans, the visceral AT is responsible for this [34,35]. If such expression leads to increased protein formation, then an increase in the size of nuchal AT depot could potentially contribute more to the total body inflammatory status. In humans, elevated inflammatory cytokines such as TNF-α, IL-6, and IL-1 play important roles in the development of obesity-associated IR [3,11,36]. If this is also the case in horses, it would further confirm the link between a high cresty neck score and IR.

Lower adiponectin mRNA expression in the SC region compared to the abdominal and nuchal region suggests that abdominal AT and nuchal AT may be more important for circulating adiponectin concentrations. Differences between gene expression in the different AT depots and the protein levels in the blood can be caused by differences at the translation level, which can be influenced by cytokines. Bruun and colleagues [37] showed that TNF-α and IL-6 significantly decreased the human adiponectin mRNA levels in vitro suggesting that endogenous cytokines may affect adiponectin. In the present study, however, no correlations between adiponectin gene expression and cytokine mRNA expression were found.

Chemotactic cytokine CCL5 mediates chemotaxis of different leukocytes, depending on the tissue protein levels. High levels of CCL5 can trigger cytokine release. In humans, CCL5 production is upregulated by inflammatory cytokines, such as IL-1 [38]. In the present study, CCL5, IL-10, and IL-1β mRNA expression was higher in the abdominal region, although no correlations between these cytokines were found. This could indicate that in horses, other cytokines regulate CCL5 production. Higher mRNA expression of CCL5, IL-10, and IL-1β in the abdominal AT suggests that this AT depot may be more important for the circulating levels of these cytokines. This is in contrast with the findings from Burns and coworkers [24] who found higher IL-1β mRNA expression in the nuchal ligament AT compared with the other depots sampled in that study. It has been suggested that different reference genes should be tested in each study setup to find the most suitable one not influenced by the experimental treatment [39]. As candidate reference gene selection was different in the present study (*HPRT1, RPL32,* and *GAPDH)* and the Burns’ study (β*-actin* and β_*2*_*-microglobulin*), it may mean that results from both studies cannot be simply compared.

In mice, many MMPs are expressed by AT and stromal vascular cells in a depot-specific manner [40]. Higher MMP-2 mRNA expression in the SC AT suggests that in horses, this AT depot is more stimulated to differentiate pre-adipocytes into adipocytes [10,41] and extracellular matrix remodeling [11].

In conclusion, different mRNA expression of inflammation-related genes in different AT depots suggests that AT depots may be a driving force for total body inflammation. It is possible that if more fat is deposited in an AT depot with high mRNA expression levels of pro-inflammatory cytokines, such as nuchal AT with high leptin and pro-inflammatory IL-6 mRNA expression, this may contribute to a greater overall inflammation in that individual horse than if the fat had been deposited in an AT depot with a low mRNA expression of pro-inflammatory cytokines. Further research into the final translation of mRNA expression of inflammation-related genes into adipokines will be necessary to correctly evaluate the impact of fat deposition at specific places in the horse body.

### Histology and immunohistochemistry

To the authors’ knowledge, adipocyte size and area in cross-section in different AT regions in the horse body have not been previously reported.

Adipocyte area and number of antigen presenting cells (APC)/adipocyte could not be determined in 10 samples from different AT depots in 6 different horses due to technical cutting and staining difficulties (N ¼ AT for horse number 4; N ¾ AT for horse number 8; tail head AT for horse number 3; mesenteric AT for horse number 3,4,7,8 and right kidney AT for horse number 2,7,9). Negative controls using isotype-matched nonsense antibody showed no staining.

**Figure 1:**
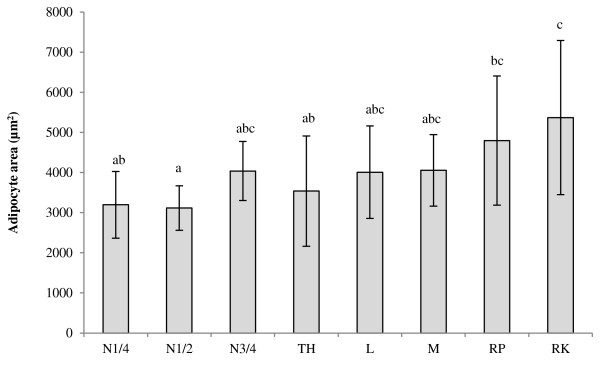
**Mean adipocyte area of equine adipocytes.** Data are reported as means ± SD. Peri-renal adipocyte area is higher compared to N ½ (P < 0.001), N ¼ (P = 0.003) and loin adipocyte area (P = 0.022). Retroperitoneal adipocyte area is higher than N ½ (P = 0.020) adipocyte area. Superscripts (abc) indicate differences between adipose tissue location (P < 0.05). Missing values and outliers were excluded from this analysis (N3/4 8; N1/4 4; TH 3; M 3,4,7,8; RK 2,7,9; L 11, RP 3). Abbreviations: N ¼, neck ¼; TH, tail head; L, loin; M, mesenteric; RP, retroperitoneal; RK, right kidney.

**Figure 2:**
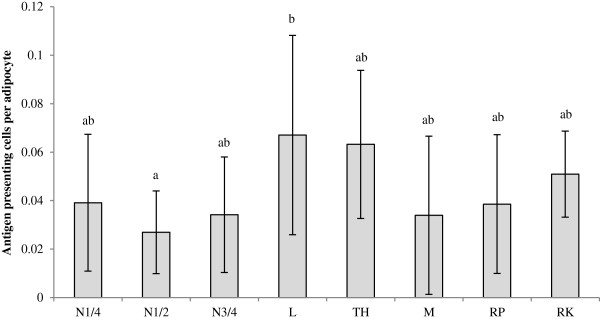
**Antigen presenting cells (APC) per adipocyte in equine adipose tissue.** Major Histocompatibility Complex II (MHC II) stain was performed to enable the identification of antigen presenting cells/HPF (APC/HPF) in the different AT depots. Data are reported as means ± SD. A lower amount of APC/adipocyte was found in the N ½ AT compared to the loin AT (0.024). Superscripts (ab) indicate differences between adipose tissue location (P < 0.05). Missing values and outliers were excluded from this analysis (N3/4 8; N1/4 4; TH 3; M 3,4,7,8; RK 2,7,9; L 11, RP 3). Abbreviations: N ¼, neck ¼; L, loin; TH, tail head; M, mesenteric; RP, retroperitoneal; RK, right kidney.

Average adipocyte size was 70 ± 7 μm, with the largest average adipocyte diameter being found in peri-renal AT (82 ± 14 μm). Average adipocyte area in cross-section was 3980 ± 1355 μm^2^. Peri-renal adipocyte area (5370 ± 1919 μm^2^) was significantly higher compared to N ½ (3116 ± 556 μm^2^; P < 0.001), N ¼ (3195 ± 831 μm^2^, P = 0.003) and tail head adipocyte area (3537 ± 1375 μm^2^, P = 0.022) (Figure 1). Retroperitoneal adipocyte area (4795 ± 1610 μm^2^) was significantly higher than N ½ (3116 ± 556 μm^2^; P = 0.020) adipocyte area. A significant lower number of APC/adipocyte was found in the N ½ AT compared to the loin AT (P = 0.024) (Figure 2).

**Figure 3:**
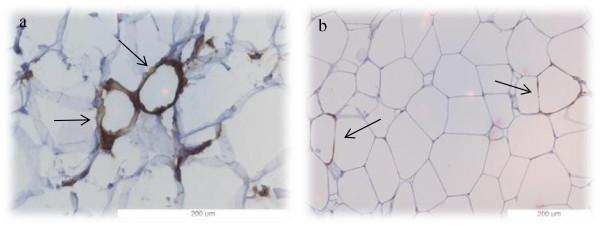
**Capping structures in horse adipose tissue.** Major Histocompatibility Complex II (MHC II) stain was performed to enable the identification of antigen presenting cells in the different AT depots. Capping structures in the loin adipose tissue **(a)** and Neck ¼ **(b)** are indicated with black arrows.

Capping structures (dead adipocytes engulfed by APC) (Figure 3), similar to CLS (dead adipocytes surrounded by macrophages [17]) in mice), were found in 6 horses in 4 different AT depots (N ¼, N ½, loin, and peri-renal). In 2 horses (number 3 and 4) CLS were found in multiple AT depots.

Clear differences in adipocyte area between AT depots were found, which is in accordance with the findings in other species [42-44]. Differences in adipocyte area can influence mRNA expression [45-47]. Significant correlations between mRNA expression of inflammation-related genes and adipocyte area were also found in the present study between multiple AT depots (N ¼, N ¾, tail head, mesenteric, retroperitoneal, and perirenal) and multiple genes (adiponectin, CCL5, SOD, IL-10, leptin, and MMP2), although no clear pattern could be determined.

In the subcutaneous region, APC/adipocyte was high compared to the nuchal and abdominal region. In humans, this is related to high adipocyte death and CLS formation [13], but in the current study, this relationship was not found. Macrophage inflammation in AT is also correlated with inflammation in humans [48,49].

This is the first study describing capping structures (adipocytes surrounded by APC) in the horse (Figure 3), probably similar to CLS in mice and humans [13,17]. One limitation of the present study was the staining method used, as this labeled MHC II molecules, which are not exclusively expressed on macrophages, but also on other APC such as monocytes, B-cells, and dentritic cells [50,51].

## Conclusions

Despite the fact that mRNA levels of inflammation-related genes were studied instead of protein levels, still interesting conclusions concerning the deposition of fat in various depots in horses could be drawn. The inflammatory profile in AT clearly varies with its location in the horse’s body in horses of different breeds varying in age and nutritional status, suggesting that the total inflammatory status of the horse may be at least partly a reflection of the relative contribution of each AT. The factors driving the interindividual differences in AT distribution thus warrant further investigation.

## Methods

### Study animals

Twelve horses, due to be euthanized for non-research purposes, were selected at a local abattoir. Horses were chosen so that : 1) they were all geldings to exclude potential gender-related gene expression [52]; 2) they represented a variety of different breeds presented at the abattoir (Table 1); 3) they showed no obvious lameness or overt laminitic rings; 4) they all had healthy appearance; 5) they ranged in their nutritional status (as scored visually by 2 experienced veterinarians) from normal to obese (Table 1); and 6) they ranged in age from 1–25 years (Table 1). Horses were euthanized according to the procedure Royal Decree of January 16, 1988 concerning the protection of animals at killing.

### Sample collection

Blood samples were taken immediately post mortem from the vena jugularis for the analysis of glucose (Vacuette® tube, FX Sodium Fluoride/Potassium oxalate, 2 ml), as well as insulin and leptin (Vacuette® tube, Z Serum Clot Activator, 9 ml). Within 15 minutes after euthanasia, AT samples were collected from the different sites: three samples in the nuchal region taken at ¼, ½, and ¾ of the distance between the poll and the withers; three abdominal samples taken at the right kidney, retroperitoneally 10 cm lateral to the linea alba, and at the mesenterium; and two samples from SC AT taken at the level of the loin and around the tail head. All samples were taken in duplicate. Gene expression samples (thickness 0.4 – 0.5 cm) were immediately submerged in RNA*later* (Sigma-Aldrich, AMBION, Inc., Austin, Texas, USA) for RNA preservation and stored at 4°C for 24 hours and then stored at -20°C until RNA extraction. Histology AT samples were stored in formalin until further processing.

### Blood sample analysis

Plasma glucose analysis was performed using a spectrophotometric method based on glucose hexokinase [53] (Architect C16000; Abbott, Abbott Laboratories, Abbott Park, Illinois, USA). Serum insulin concentrations were measured with an immunoradiometric assay test kit [32] (insulin IRMA Ref 5251, Diasource Europe S.A., Nivelles, Belgium). An implementation validation has been carried out before use in horses. A dilution curve has been designed (100–80 – 60 – 40 – 20 – 0% sample). Theoretical and measured values were compared to evaluate possible matrix-influences. Inter-assay variance was < 4%, intra-assay variance in the high sample% was 9.2%, in the low sample% 1.9%. Leptin was measured using a multispecies RIA kit (Merck Millipore., Billerica, MA 01821, USA), previously validated for use in equine plasma [54].

### RNA isolation and cDNA synthesis

Total RNA was isolated using the RNeasy Lipid Tissue Mini Kit (Qiagen®, AMBION, Inc., USA) and the TissueRuptor (Qiagen) for complete sample disruption/homogenization, as described in the manufacturer’s protocol. An on-column DNase digestion (RNase-Free DNase Set, Qiagen) was included and was empirically verified by a minus reverse transcription (RT) control reaction. RNA quantity and purity (OD 260/280 ratio 1.9-2.1) were measured with the ND-1000 spectrophotometer (NanoDrop, NanoDrop Products, Wilmington, USA). The RNA quality was verified on an agarose gel and was assessed with the Ex-on RNA StdSens Analysis Kit (Bio-Rad, Bio-Rad Laboratories N.V., Hercules, USA) on an Experion Automated Electrophoresis System (Bio-Rad). The RNA quality indicator (RQI) for the AT ranged between 7–8.5 and for liver between 9–9.5. Subsequently, the iScript cDNA synthesis kit (Bio-Rad) was used to convert approximately 0.6 μg of total RNA into cDNA, which was verified by a control PCR.

### Quantitative real-time PCR

All PCR reactions were performed in a 15 μl reaction volume on an iCycler iQ Real-Time PCR Detection System (Bio-Rad) using 7.5 μl of Kapa SYBR Fast Bio-Rad qPCR Master Mix (Sopachem, Kapabiosystems, Woburn, USA) supplemented with 2.5 μl of diluted cDNA. The addition of RNAse free water and primer concentration varied according to the primer used. The qRT-PCR measurements for all samples were performed in duplicate and every run included a no-template control.

The PCR program started with an initial denaturation at 95°C for 3 minutes to activate the *Taq* polymerase, followed by 40 cycles of denaturation at 95°C for 10 seconds and a combined primer annealing/extension at the primer specific annealing temperature for 30 seconds during which fluorescence was measured. A melting curve was constructed to verify the presence of a single gene-specific amplicon and the absence of any primer dimers by heating the samples from 70 to 95°C in 0.5°C increments with a dwell time at each temperature of 10 seconds while continuously monitoring the fluorescence. The efficiency of each RT-qPCR run was calculated from a relative standard curve based on a 5-point 5-fold cDNA dilution series using pooled cDNA obtained from AT in the neck, loin and tail head region and liver. The RT-qPCR data from all genes were converted to raw data as described in Erkens *et al.*[31]. Six candidate reference genes were selected based on previous gene expression studies in human [30,55-57] and bovine AT [58], as well as equine tissues (skin, blastocysts, and lymphocytes) [59-61]. Candidate reference gene primers for *ACTB*, *HPRT1*, *RPL32,* and *TUBA4A* were used from Bogaert *et al.* (2006) [59]; *GAPDH* and *SDHA* were used from Smits *et al.*[60].

### Determination of candidate reference gene expression stability

Candidate reference gene expression stability was evaluated with the M value of the geNorm algorithm [62]. The most stable control genes (lowest variation in mRNA expression) have the lowest M value. The raw gene expression data from the genes of interest were then normalised using the geometric mean of the best performing candidate reference genes.

### Primers for the genes of interest

Primers for the genes of interest *CCL5*, *IL-10*, *IL-1*β*, IL-6,* and *SOD2* were used from Figueiredo *et al.*[63], and for *MMP2* from Loftus *et al.*[64]. For *ADIPOQ* and *LEP*, primers were designed using Primer3 [65]*,* while taking primer specificity (Blast, [66]) and possible secondary structures (Mfold, [67]) into account. As for the candidate reference genes, all primer amplicons were sequenced using the BigDye Terminator v3.1 Cycle Sequencing Kit (Applied Biosystems) on an Applied Biosystems 3730xl DNA Analyser. In addition, gel electrophoresis was performed to check the formation of 1 amplicon of the expected size, and to control the absence of primer dimers.

### Histology

Adipose tissue samples were fixated by immersion in 4% paraformaldehyde, embedded in paraffin and sectioned. Two five μm thick serial sections were obtained, the first stained by hematoxylin and eosin (HE) to assess morphology (adipocyte area) and the rest processed for immunohistochemistry (see below). Adipocyte numbers were counted in 10 high power fields (HPF) and the average number of adipocytes was calculated per HPF. The surface of 1 HPF (πr^2^ = π*250 μm^2^ = 196250 μm^2^) was divided through the numbers of adipocytes/HPF to calculate the mean surface area per adipocyte.

### Immunohistochemistry

The presence of antigen presenting cells (APC) was evaluated by the use of Monoclonal Mouse Anti-Human HLA-DR antigen, alpha-chain clone TAL.1B5 (Code No. M0746; DakoCytomation, DakoCytomation, DK-2600, Glostrup, Denmark). This stain colours the major histocompatibility II (MHC II) molecules that are expressed on cells that serve as APC for CD4+, such as macrophages, monocytes, dendritic cells, and B cells [51,52]. Five μm-thick paraffin-embedded sections mounted on coated slides (APES, 3-aminopropyltriethoxysilane) were deparaffinised in xylene and with ethanol. Subsequently, the slides were pre-treated according to the microwave pressure cooker protocol for antigen retrieval (Citrate Buffer 10x, pH 6.0, Klinipath CBB 999, Klinipath BV, 6920 AD, Duiven, Netherlands). The immunohistochemistry was performed in an automated immunostainer (Dako, Glostrup, Denmark; S/N S38-7410-01) according to the manufactures protocol. For visualization the Envision+/HRP mouse (DAB) kit (Dako Ref K4007, DakoCytomation, DK-2600, Glostrup, Denmark) was used. Antibody diluent (Dako Ref S302283) was used to block hydrophobic interactions. Sections were counterstained with Mayer’s hemalum solution (Klinipath). A positive control (thoracic mass, high grade sarcoma) was included in each run to ensure specificity. In negative controls, the primary antibody was replaced by a nonsense antibody of similar isotype (Monoclonal Mouse Anti-Human Cytokeratin Clones AE1/AE3). A second type of negative control was carried out by using central nervous system (CNS) parenchyma with the original primary antibody (Monoclonal Mouse Anti-Human HLA-DR antigen (alpha-chain clone TAL.1B5), as within the normal CNS parenchyma, MHC expression is minimal or absent [68]. To calculate APC/adipocyte, number of APC/10 high HPF was divided through the number of adipocytes/10 HPF.

### Statistical analysis

Data are reported as means ± SD and significance was set at P < 0.05. Statistical analyses were performed using IBM SPSS Statistics 20. Gene expression data were analysed with a general linear model by means of repeated measures with depot as within variable and animal as between variable, followed by a Bonferroni post hoc test when a significant difference between depots was detected. Histology data were analysed with a general linear model, univariate analysis and Tukey post hoc test. Correlation analysis (Pearson’s correlation test) was performed to identify relationships between blood parameters, histology findings and mRNA expression of cytokines.

## Abbreviations

AT: Adipose tissue; RT-qPCR: Quantitative real-time polymerase chain reaction; mRNA: Messenger ribonucleic acid; ACTB: Beta actin; HPRT1: Hypoxanthine phosphoribosyltransferase 1; TUBA4A: Tubulin; RPL 32: Ribosomal protein L32; GAPDH: Glyceraldehyde-3-phosphate dehydrogenase; SDHA: Succinate dehydrogenase complex; IL-10: Interleukin 10; IL-6: Interleukin 6; IL-1β: Interleukin 1β; TNF-α: Tumor necrosis factor α; CCL5: Chemokine ligand 5; MMP2: Matrix metalloproteinase 2; CLS: Crown like structure; SC: Subcutaneous; IR: Insulin resistance; cDNA: Complementary deoxyribonucleic acid; Cq: Transcription value; N ¼: Neck ¼; SOD: Superoxide dismutase; APC: Antigen presenting cell; HE: Hematoxylin and eosin; HPF: High power field; MHC II: Major histocompatibility complex II; APES: 3-aminopropyltriethoxysilane.

## Competing interests

The authors declare that they have no competing interests.

## Authors’ contributions

LB was the primary author of the manuscript, responsible for the study design and performed most of the study procedures. TE participated in the study procedures and provided real-time instrument procedures. LJP, RD, GPJJ, PAH, and MH participated in the design of the project, helped to draft the manuscript and supervised the study. All authors read and approved the final manuscript.
